# Alx4 relays sequential FGF signaling to induce lacrimal gland morphogenesis

**DOI:** 10.1371/journal.pgen.1007047

**Published:** 2017-10-13

**Authors:** Ankur Garg, Mukesh Bansal, Noriko Gotoh, Gen-Sheng Feng, Jian Zhong, Fen Wang, Ariana Kariminejad, Steven Brooks, Xin Zhang

**Affiliations:** 1 Departments of Ophthalmology, Pathology and Cell Biology, Columbia University, New York, NY, United States of America; 2 Department of Biochemistry and Molecular Biology, Indiana University School of Medicine, Indianapolis, IN, United States of America; 3 PsychoGenics Inc., Tarrytown, NY, United States of America; 4 Division of Cancer Cell Biology, Cancer Research Institute, Kanazawa University Kakuma-machi, Kanazawa city, Japan; 5 Department of Pathology, School of Medicine, and Section of Molecular Biology, Division of Biological Sciences, University of California San Diego, La Jolla, CA, United States of America; 6 Burke Medical Research Institute, Feil Family Brain and Mind Research Institute, Weill Cornell Medicine, White Plains, NY, United States of America; 7 Center for Cancer Biology and Nutrition, Institute of Biosciences and Technology, Texas A&M, Houston, TX, United States of America; 8 Kariminejad-Najmabadi Pathology & Genetics Center, Tehran, Iran; National Cancer Institute, UNITED STATES

## Abstract

The sequential use of signaling pathways is essential for the guidance of pluripotent progenitors into diverse cell fates. Here, we show that Shp2 exclusively mediates FGF but not PDGF signaling in the neural crest to control lacrimal gland development. In addition to preventing p53-independent apoptosis and promoting the migration of Sox10-expressing neural crests, Shp2 is also required for expression of the homeodomain transcription factor Alx4, which directly controls *Fgf10* expression in the periocular mesenchyme that is necessary for lacrimal gland induction. We show that *Alx4* binds an *Fgf10* intronic element conserved in terrestrial but not aquatic animals, underlying the evolutionary emergence of the lacrimal gland system in response to an airy environment. Inactivation of *ALX4/Alx4* causes lacrimal gland aplasia in both human and mouse. These results reveal a key role of Alx4 in mediating FGF-Shp2-FGF signaling in the neural crest for lacrimal gland development.

## Author summary

The dry eye disease caused by lacrimal gland dysgenesis is one of the most common ocular ailments. In this study, we show that Shp2 mediates the sequential use of FGF signaling in lacrimal gland development. Our study identifies *Alx4* as a novel target of Shp2 signaling and a causal gene for lacrimal gland aplasia in humans. Given this result, there may also be a potential role for *Alx4* in guiding pluripotent stem cells to produce lacrimal gland tissue. Finally, our data reveals an Alx4-Fgf10 regulatory unit broadly conserved in the diverse array of terrestrial animals from humans to reptiles, but not in aquatic animals such as amphibians and fish, which sheds light on how the lacrimal gland arose as an evolutionary innovation of terrestrial animals to adapt to their newfound exposure to an airy environment.

## Introduction

The lacrimal gland plays an essential role in protecting the ocular surface by secreting the aqueous components of the tear film. Defects associated with the lacrimal gland are the main cause of dry eye disease, which is highly prevalent in the geriatric population [1]. Left untreated, dry eye disease may progress from eye irritation and corneal scarring to eventual vision loss. However, lacrimal gland dysfunction is currently incurable and the common treatment option for the resulting dry eye pathology is the application of artificial tears that provides only temporary relief. Recent studies have shown that engraftment of lacrimal gland germ can restore lacrimation in animal models, but the procurement of lacrimal gland cells remains an unresolved challenge [2]. A better fundamental understanding of lacrimal gland development may inform cell-based therapies to repair or regenerate the lacrimal gland, which holds great promise for the treatment of dry eye disease [3].

The neural crest is a multipotent stem cell population that gives rise to many diverse tissues, including craniofacial bones and cartilage, smooth muscle, neurons and ganglia of the peripheral nervous system, adipose cells and melanocytes [4, 5]. Upon induction at the neural plate border, the neural crest undergoes an epithelial-to-mesenchymal transition to delaminate from the dorsal neural tube. These cells then migrate to different regions of the embryo and differentiate into distinct cell types, guided by both their origins along the anterior-posterior axis and the signaling cues they are exposed to in their immediate environment [6]. Once at their destination, neural crest cells closely interact with their host organs, influencing their patterning and morphogenesis [7]. The cranial neural crest cells originating from the midbrain are the source of the periocular mesenchyme, which expresses the chemoattractive signal of Fgf10 to regulate lacrimal gland development [8, 9]. By binding to Fgfr2b and heparan sulphate proteoglycan co-receptors, Fgf10 induces the invasion and branching of the lacrimal gland epithelium [8, 10, 11]. This essential role of Fgf10 in branching morphogenesis is conserved in glandular organs that include the lung, prostate, and pancreas. Nonetheless, the control of Fgf10 expression in the neural crest derived tissues remains unknown.

In this study, we showed that FGF signaling mediated by the protein phosphatase Shp2 is required for the proper patterning and differentiation of the neural crest-derived mesenchyme to produce Fgf10. Genetic evidence further demonstrates that Shp2 is recruited by Frs2 to activate Ras-MAPK signaling downstream to Fgfr1 and Fgfr2 but not to Pdgfrα in the neural crest. By differential gene expression analysis, we identified the homeodomain transcription factor Alx4 as the key effector of Shp2 signaling to control the expression of Fgf10 in the periocular mesenchyme. Importantly, the Alx4 binding sequence in the *Fgf10* gene locus is conserved in land species from human to lizard, but not in aquatic animals such as frog and fish, which provides a new genetic insight into how the lacrimal gland arose as an evolutionary innovation of terrestrial animals to adapt to the dry environment. *Alx4* conditional knockouts disrupted lacrimal gland development in mouse models and a homozygous *ALX4* mutation causes lacrimal gland aplasia in human. Our results reveal a FGF-Shp2-Alx4-Fgf10 axis in regulating neural crest and lacrimal gland development.

## Results

### Lacrimal gland development requires FGF but not PDGF signaling in the neural crest

**Fig 1:**
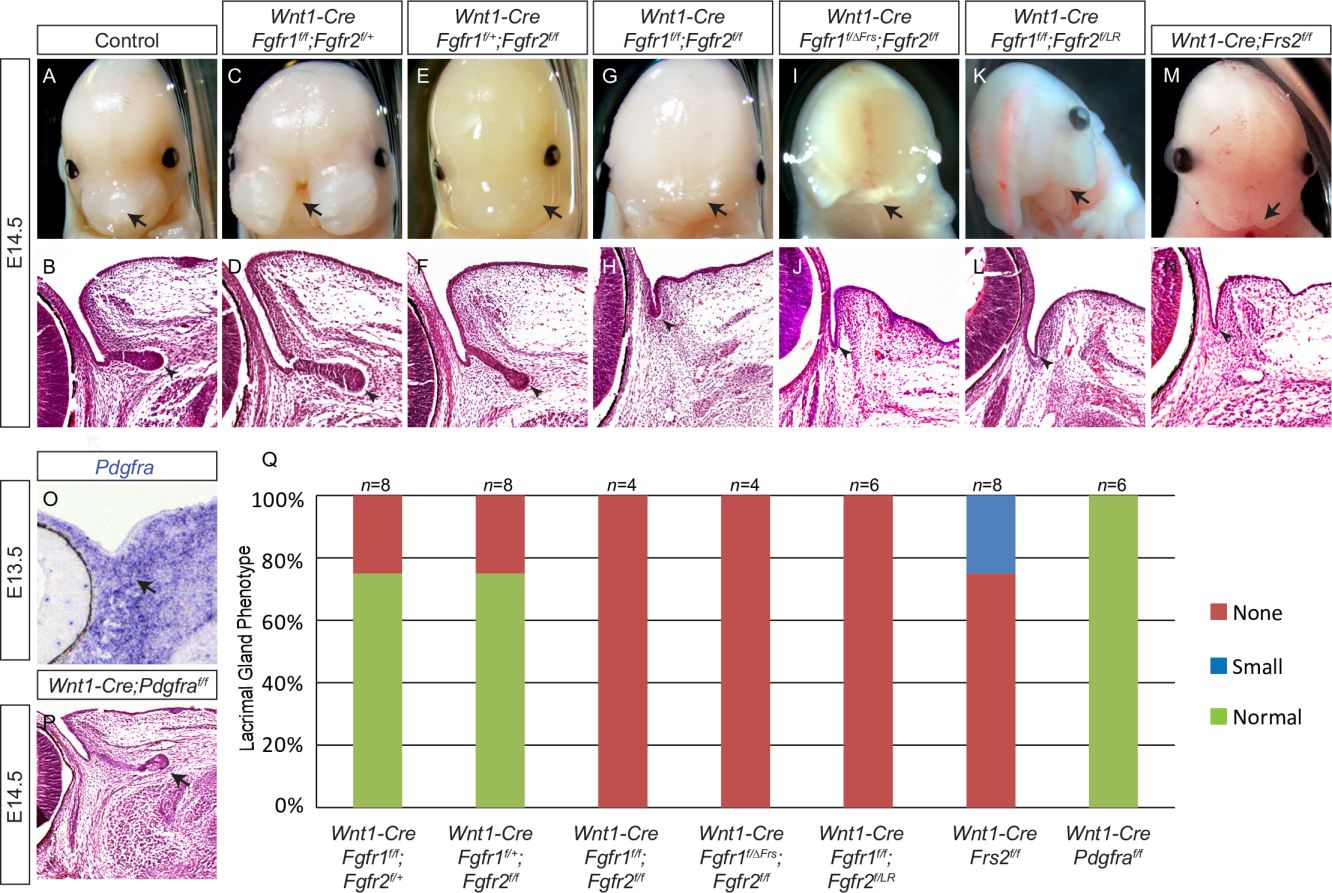
The neural crest specific ablation of *Fgfr* and *Frs2* disrupted lacrimal gland development. (**A-N**) Lacrimal gland budding occurred in *Fgfr1* and *Fgfr2* single, but not in double, mutants (A-H, arrowheads). A mutation of the Frs2 binding site on *Fgfr1* (*Fgfr1*^*ΔFrs*^) or *Fgfr2* (*Fgfr2*^*L/R*^), or the deletion of *Frs2* altogether resulted in the disruption of lacrimal gland development (I-N, arrowheads). Note that the severity of the craniofacial phenotype does not always correlate with the defects present in the lacrimal gland (compare C, D, M and N). Arrow: craniofacial abnormalities. Arrowheads: lacrimal gland primordia. e: eye. (**O-P**) Although *Pdgfra* was expressed in the periocular mesenchyme (O, arrow), its deletion in the neural crest did not affect lacrimal gland budding (P, arrow). (**Q**) Quantification of lacrimal gland phenotype.

FGF signaling is important for development of the neural crest derived craniofacial structures [12–18]. Using the neural crest specific *Wnt1-Cre*, we observed that conditional knockout of *Fgfr1* resulted in significant craniofacial abnormalities, whereas deletion of *Fgfr2* did not exhibit any obvious effect (Fig 1A, 1C and 1E, arrows). Lacrimal gland development begins with the invasion of an epithelial bud from the conjunctiva into the periocular mesenchyme at embryonic day 14.5 (E14.5) (Fig 1B, arrowhead). In *Fgfr1* and *Fgfr2* single mutants, lacrimal gland development was mostly unaffected (Fig 1D and 1F, arrowheads). Combined deletion of both *Fgfr1* and *Fgfr2*, however, abrogated lacrimal gland budding (Fig 1G and 1H, arrows), indicating that Fgfr1 and Fgfr2 can compensate for each other in the neural crest during lacrimal gland development. *Fgfr1*^*ΔFrs*^ and *Fgfr2*^*LR*^ alleles encode the mutants Fgfr1 and Fgfr2 that lack the docking site for the adaptor protein Frs2 [16, 19]. Although *Fgfr2*^*LR*^ homozygous mice were viable and fertile, the craniofacial and lacrimal gland mutant phenotypes were observed in both the *Wnt1-Cre;Fgfr1* ^*f/ΔFrs*^;*Fgfr2* ^*f/f*^ and *Wnt1-Cre;Fgfr1* ^*f/f*^;*Fgfr2* ^*f/LR*^ mutants (Fig 1I–1L, arrows). The essential role of Frs2 in the neural crest for lacrimal gland development was further demonstrated in *Wnt1-Cre;Frs2*^*f/f*^ mutants, which displayed a less severe craniofacial phenotype than *Fgfr* mutants, but a similar cessation of lacrimal gland budding (Fig 1M and 1N, arrows). Finally, lacrimal gland development was also aborted in *Wnt1-Cre;Frs2*^*f/2F*^ animals, which carried mutations in two tyrosine residues of Frs2 (*Frs2*^*2F*^) required for the binding of the Shp2 protein phosphatase (S1 Fig, *n* = 6) [20]. In contrast, although *Pdgfrα* was expressed in the periocular mesenchyme and required for craniofacial development, its neural crest specific knockout failed to impair lacrimal gland development (Fig 1O–1Q, arrows). These results demonstrated that lacrimal gland development specifically requires FGF-Frs2-Shp2 signaling in the neural crest.

### Neural crest Shp2 regulates *Fgf10* expression in the periocular mesenchyme for lacrimal gland development

**Fig 2:**
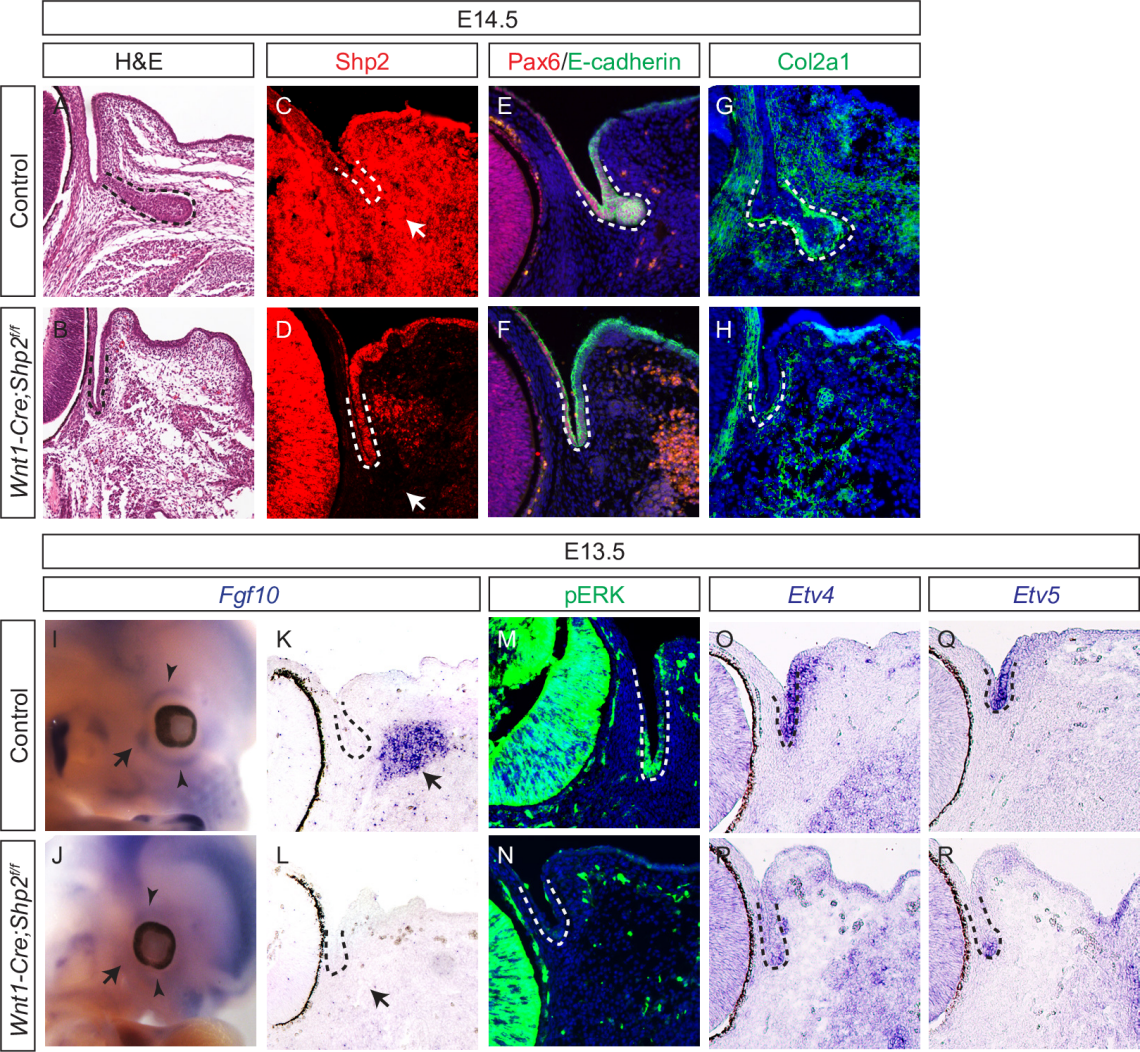
Lacrimal gland budding requires *Shp2* in the neural crest. (**A-H**) *Wnt1-Cre* mediated ablation of *Shp2* in the neural crest resulted in the complete loss of Shp2 staining within the periocular mesenchyme (C and D, arrows). This consequently lead to the abrogation of the lacrimal gland buds that are normally present at E14.5 (A-D, dotted lines). The lacrimal gland primordia in *Shp2* mutants still expressed Pax6 and E-cadherin (E-F, dotted lines), but failed to upregulate Col1a1 expression (G-H, dotted lines). (**I-R**) At E13.5, *Fgf10* is normally expressed in the periocular mesenchyme to induce pERK, *Etv4* and *Etv5* in the lacrimal gland bud, but these downstream targets were all down regulated in the *Shp2* mutants. Arrow: *Fgf10* expression near to the future lacrimal gland bud. Arrowhead: *Fgf10* expression in the eyelid mesenchyme. The lacrimal gland primordia were outlined with dotted lines.

To investigate the potential downstream targets of neural crest FGF signaling occurring during lacrimal gland development, we next generated *Wnt1-Cre;Shp2*^*f/f*^ mutants, which failed to develop a lacrimal gland as expected (Fig 2A and 2B, dotted lines, *n* = 6). Consistent with the idea that the neural crest is the main contributor of the periocular mesenchyme, immunostaining confirmed that Shp2 protein was depleted in the periocular mesenchyme, but preserved in the ectoderm-derived conjunctival epithelium (Fig 2C and 2D, arrows and dotted lines). Although the epithelial cells maintained Pax6 and E-cadherin staining, there was no increase in Col2a1 expression, a hallmark of the nascent lacrimal gland bud (Fig 2E–2H, dotted lines). By contrast, the periocular mesenchyme expression of Col2a1 was preserved, suggesting that the identity of these neural crest-derived cells was unchanged. The *Wnt1-Cre* transgene was recently reported to cause ectopic expression of *Wnt1* in the midbrain-hindbrain boundary [21]. To ensure that this complication did not compromise our results, we used another neural crest-specific deletor, *Sox10-Cre*, to ablate *Shp2*, which also resulted in the dysgenesis of the lacrimal gland (S2A and S2B Fig, arrows). Altogether, these results show that Shp2 signaling in the neural crest is required for lacrimal gland budding in a non-cell autonomous manner.

The initial budding of the lacrimal gland requires the inductive signal of Fgf10 that emanates from the periocular mesenchyme. In E13.5 control embryos, *Fgf10* was found to exist in a ring-type pattern along the presumptive eyelid surrounding the eye (Fig 2I, arrowheads), with the strongest signal occuring in the mesenchyme adjacent to the future lacrimal gland bud (Fig 2I and 2K, arrows). In both *Wnt1-Cre;Shp2*^*f/f*^ and *Sox10-Cre;Shp2*^*f/f*^ mutants, however, *Fgf10* was absent in the entire periocular mesenchyme (Fig 2J and 2L, arrows and arrowheads, and S2C and S2D Fig). As a result, ERK phosphorylation was maintained in the adjacent retina but abolished in the conjunctival epithelium (Fig 2M and 2N, dotted lines), suggesting a specific loss of FGF signaling in the lacrimal gland primordia. This evidence was further supported by the observed down regulation of FGF signaling response genes, *Etv4* and *Etv5*, in the presumptive lacrimal gland epithelium (Fig 2O–2R, dotted lines). Considering the essential role of Fgf10 signaling in inducing lacrimal gland budding, we concluded that the absence of *Fgf10* expression accounted for the lacrimal gland aplasia seen in neural crest *Shp2* mutants.

### Ras-MAPK signaling and ETS transcription factors are downstream effectors of Shp2

**Fig 3:**
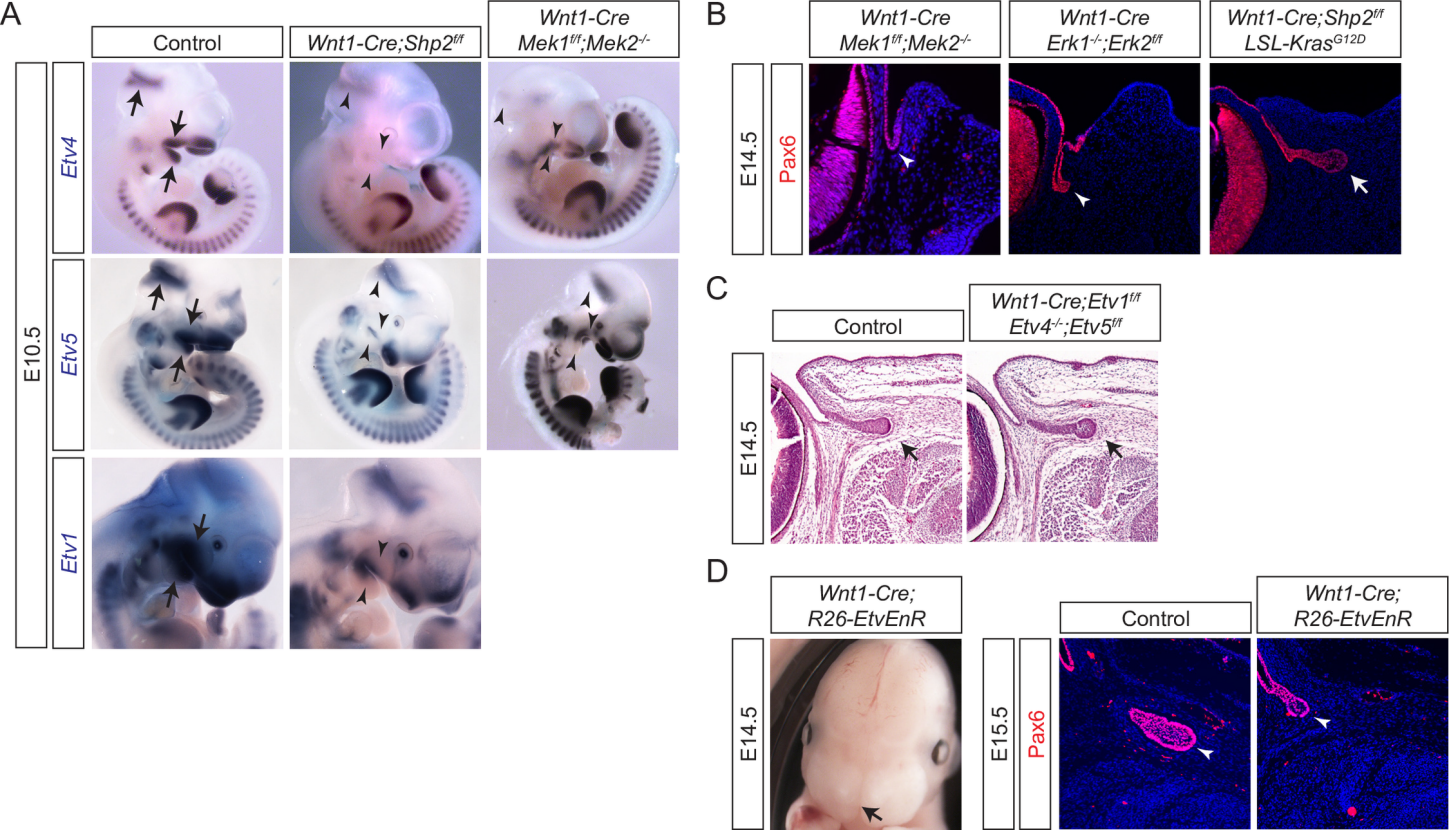
Shp2 regulates MAPK-Etv signaling in the neural crest. (**A**) FGF signaling target genes *Etv1*, *Etv4* and *Etv5* were expressed in the midbrain-hindbrain junction, branchial arches and nasal placode. These expressions patterns were significantly reduced by the deletions of *Shp2* and *Mek1/2* in the neural crest. Arrows point to *Etv*-expressing regions in the brain. (**B**) Lacrimal gland budding was lost in *Wnt1-Cre; Mek1*^*f/f*^*;Mek2*^*-/-*^ and *Wnt1-Cre; Erk1*^*-/-*^*;Erk2*^*f/f*^ mutants, but rescued by the constitutive activation of Ras signaling in *Wnt1-Cre;Shp2*^*f/f*^*;LSL-Kras*^*G12D*^ embryos. Arrow: lacrimal gland primordia. (**C**) *Wnt1-Cre* mediated deletion of *Etv1*, *4* and *5* failed to disrupt lacrimal gland development. (**D**) Expression of the Etv4*-*Engrailed repressor (EnR) fusion protein in the neural crest led to craniofacial defects (arrow) and reduced lacrimal gland budding (arrowheads).

FGF signaling is known to activate the Ras family of small GTPases, which play important roles in cell proliferation, migration and differentiation. Previous studies have identified multiple downstream targets of Ras, including Raf kinases, type I phosphoinositide (PI) 3-kinases, Ral guanine nucleotide exchange factors, the Rac exchange factor Tiam1, and phospholipase C3 [22]. Among these molecules, Raf kinases activate the mitogen-activated protein kinase (MAPK) cascade that culminates with the phosphorylation of Mek kinases (Mek1 and 2) and their direct Erk kinase targets (Erk1 and 2) [23]. At E10.5, ETS transcription factors *Etv1*, *4* and *5* were strongly expressed in tissues known to have active FGF signaling (Fig 3A, arrows). In both *Wnt1-Cre;Shp2*^*f/f*^ and *Wnt1-Cre; Mek1*^*f/f*^*;Mek2*^*-/-*^ embryos, these expression patterns were significantly down regulated in the cranial neural crest-derived mesenchyme in the midbrain, branchial arches and nose (Fig 3A, arrowheads), supporting the claim that Shp2 and Mek operate in the same signaling cascade leading to *Etv1*, *4*, and *5 expression*. Furthermore, lacrimal gland development was never initiated after the genetic removal of *Mek1/2* in the neural crest (Fig 3B, arrowhead, *n* = 8). Interestingly, however, a small lacrimal gland protrusion was seen in *Wnt1-Cre; Erk1*^*-/-*^*;Erk2*^*f/f*^ embryos, suggesting that Mek may have additional key targets other than Erk (Fig 3B, arrowhead, *n* = 2) that participate in budding morphogenesis. Furthermore, by taking advantage of a conditional allele of oncogenic *Kras* (*LSL-Kras*^*G12D*^*)*, we showed that constitutively active Ras signaling in the neural crest rescued the *Shp2* deficiency during lacrimal gland budding (Fig 3B, arrow, *n* = 10), supporting the downstream role of Ras-MAPK activation in the FGF-Shp2 signaling cascade in the neural crest [24–27].

The faithful expression of *Etv1*, *4* and *5* in response to Ras-MAPK activity prompted us to investigate the functional significance of these three transcription factors. Surprisingly, even the combined inactivation of *Etv1/4/5* in the neural crest lineage failed to perturb lacrimal gland development (Fig 3C, *n* = 8), suggesting that these genes may be compensated by other ETS domain transcription factors that share similar binding specificity. To overcome this genetic redundancy, we used a Cre-inducible transgene (*R26-EtvEnR*) to express *Etv4* fused with the *Engrailed* repressor domain, which acts as a dominant negative ETS transcription factor [28]. *Wnt1-Cre; R26-EtvEnR* embryos not only exhibited the previously observed craniofacial defect (Fig 3D, arrow), but also showed reduced elongation of the lacrimal gland (Fig 3D, arrowheads, *n* = 8). This result suggests that ETS domain transcription factors are downstream effectors of FGF-Shp2-Ras-MAPK signaling in neural crest development.

### Lacrimal gland aplasia is not due to aberrant neural crest induction, migration or cell death

**Fig 4:**
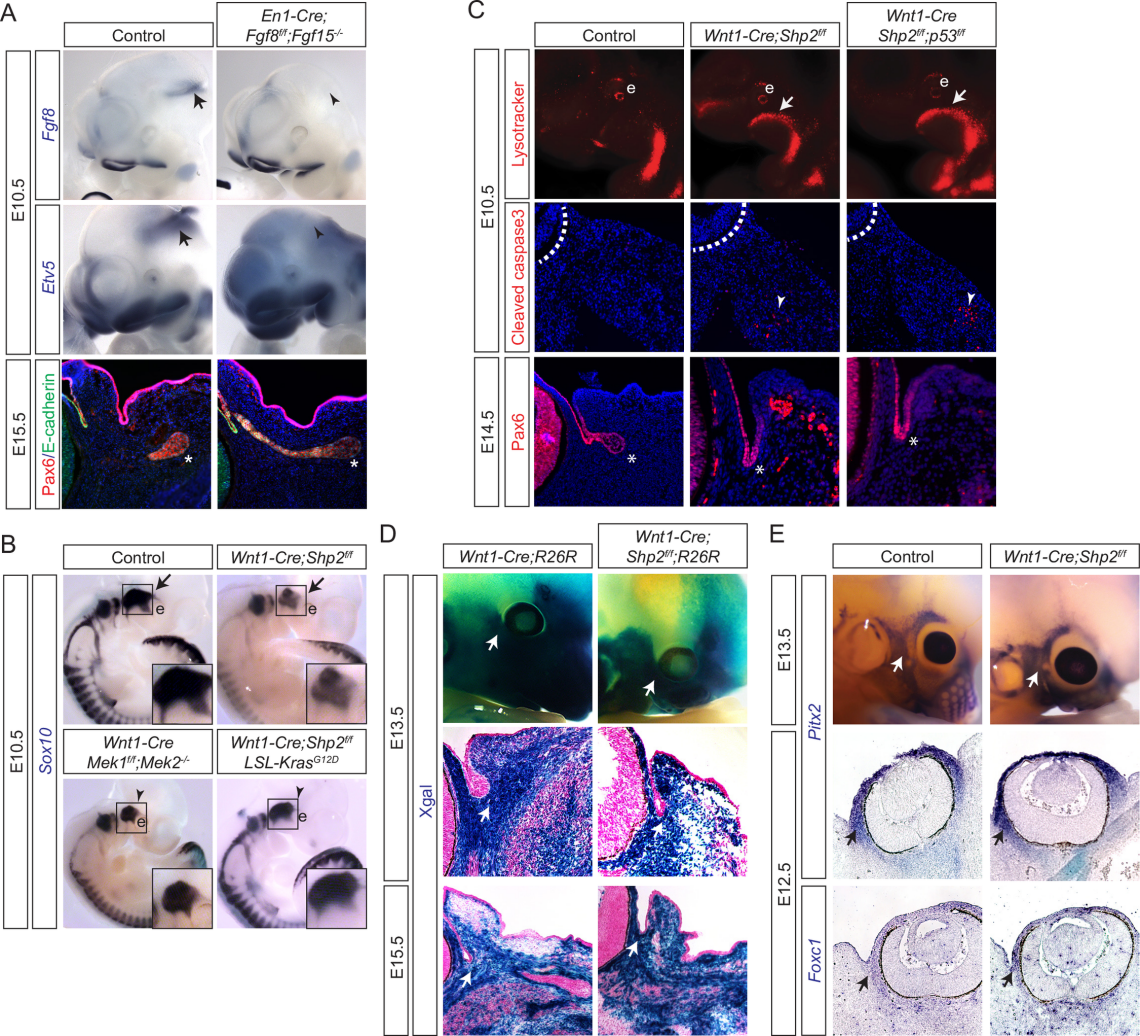
Shp2 deletion did not prevent the neural crest from giving rise to the periocular mesenchyme. (**A**) In E10.5 *En1-Cre;Fgf8*^*f/f*^*;Fgf15*^*-/-*^ embryos, *Fgf8* was ablated in the midbrain-hindbrain junction, where FGF signaling response gene *Etv5* was also down regulated, indicating a loss of FGF signaling. Nonetheless, lacrimal gland budded at E15.5 was unaffected. Arrow and arrowhead: *Fgf8* and *Etv5* expressions in the midbrain-hindbrain junction. Asterisks: lacrimal gland bud. (**B**) The migrating neural crest marked by *Sox10* expression was reduced in *Wnt1-Cre;Shp2*^*f/f*^ and *Wnt1-Cre; Mek1*^*f/f*^*;Mek2*^*-/-*^ mutants, but rescued in *Wnt1-Cre;Shp2*^*f/f*^*;LSL-Kras*^*G12D*^ embryos. Arrow and arrowhead: *Sox10* positive neural crest cells in the periocular mesenchyme. e: eye. (**C**) Deletion of *p53* in *Shp2* mutants failed to prevent aberrant apoptosis in the branchial arches and in the periocular mesenchyme shown by lysotracking (upper panel) and cleaved caspase 3 staining (middle panel), respectively. Lacrimal gland budding was not rescued in the *Shp2/p53* double mutants (bottom panel). Arrow: lysotracker staining in the branchial arch. Arrowhead: apoptotic cells in the periocular mesenchyme. Asterisk: developing lacrimal gland bud. (**D**) Lineage tracing by crossing *Wnt1-Cre* mice with *R26R* reporter mice showed that Shp2 ablation did not prevent neural crest cells from populating the periocular mesenchyme after E13.5. Arrow: Xgal-stained neural crest cells. (**E**) Periocular mesenchyme markers *Pitx2* and *Foxc1* were unperturbed in *Shp2* mutants.

FGF signaling has been implicated in the induction, proliferation, migration and differentiation of neural crest cells [13, 29–32]. The periocular mesenchyme originates from the neural tube in the midbrain, where active FGF signaling indicated by *Etv5* expression coincides with *Fgf8* expression (Fig 4A, arrows). This suggests that Fgf8 may activate FGF signaling during the induction of cranial neural crest cell progenitors. Considering that *Fgf15* is also expressed in the midbrain, we ablated *Fgf8* in the midbrain-hindbrain junction using *En1-Cre* in the *Fgf15* null background. As expected, both *Fgf8* and *Etv5* midbrain expressions were absent in *En1-Cre;Fgf8*^*f/f*^*;Fgf15*^*-/-*^ embryos (Fig 4A, arrowheads), demonstrating a loss of FGF signaling. Nevertheless, the lacrimal gland bud still developed normally in these mutants (Fig 4A, asterisks; *n* = 3), showing that FGF signaling at the induction of cranial neural crest cells is not required for lacrimal gland development.

After induction at the dorsal neural tube, the neural crest progenitors express *Sox10* as they begin to migrate toward their final destination. At E10.5, although *Sox10*-positive neural crest cells were present in the cranial mesenchyme in *Wnt1-Cre;Shp2*^*f/f*^ mutants, both their number and extent of migration were slightly reduced as compared to those in the control embryos (Fig 4B, arrows), suggesting that the loss of Shp2 produces subtle defects in neural crest proliferation and migration. This phenotype was reproduced in *Wnt1-Cre; Mek1*^*f/f*^*;Mek2*^*-/-*^ embryos, but ameliorated in *Wnt1-Cre;Shp2*^*f/f*^*;LSL-Kras*^*G12D*^ embryos (Fig 4B, arrowheads), supporting a role for Shp2-Ras-MAPK signaling in post-inductive neural crest cells.

Previous studies in zebrafish suggested that Shp2 may have a MAPK-independent function in preventing p53-mediated apoptosis in the neural crest [26]. Using lysotracker dye to stain acidic lysosomes in cells undergoing apoptosis, we observed extensive cell death in the first pharyngeal arch in E10.5 *Shp2* mutant embryos (Fig 4C, arrows). In sections, cleaved-caspase 3 staining also detected abnormal cell apoptosis in the periocular mesenchyme, although the apoptotic regions were far removed from the conjunctiva (Fig 4C, arrowheads). We reasoned that if the apoptosis induced by the *Shp2* deletion was indeed dependent on p53, then the apoptotic events may be avoided by the removal of *p53*. However, ablation of *p53* in *Shp2* mutants failed to prevent cell death in the first pharyngeal arch or to rescue any craniofacial phenotype (Fig 4C, arrows and arrowheads). Further, in lacrimal gland development, budding morphogenesis was still aborted in *Wnt1-Cre; Shp2*^*f/f*^*;p53*^*f/f*^ embryos (Fig 4C, asterisks, *n* = 6). Therefore, p53 was not responsible for either the neural crest cell death or the lacrimal gland aplasia observed in *Shp2* mutants.

To determine whether these early onset neural crest defects affect periocular mesenchyme development, we crossed *Wnt1-Cre* mice with those containing the *R26R* Cre reporter to follow the fate of the neural crest cells. Interestingly, by the time of lacrimal gland budding at E13.5, the periocular mesenchyme adjacent to the conjunctival epithelium was already occupied by the neural crest derived cells in *Shp2* mutants (Fig 4D, arrows). Furthermore, the expression of *Pitx2* and *Foxc1*, two markers of the neural crest derived periocular mesenchyme, were similar in wild-type control and *Shp2* mutant eyes (Fig 4E, arrows). Therefore, despite causing an initial delay in neural crest migration and abnormal apoptosis, *Shp2* ablation did not disrupt the occupancy of the periocular mesenchyme by the neural crest-derived cells at the time of lacrimal gland budding. We thus concluded that the subtle neural crest migration, survival and proliferation defects seen in *Shp2* mutants were unlikely to account for the complete failure of lacrimal gland development.

### Shp2 signaling regulates Alx1 and Alx4 expression in the periocular mesenchyme

**Fig 5:**
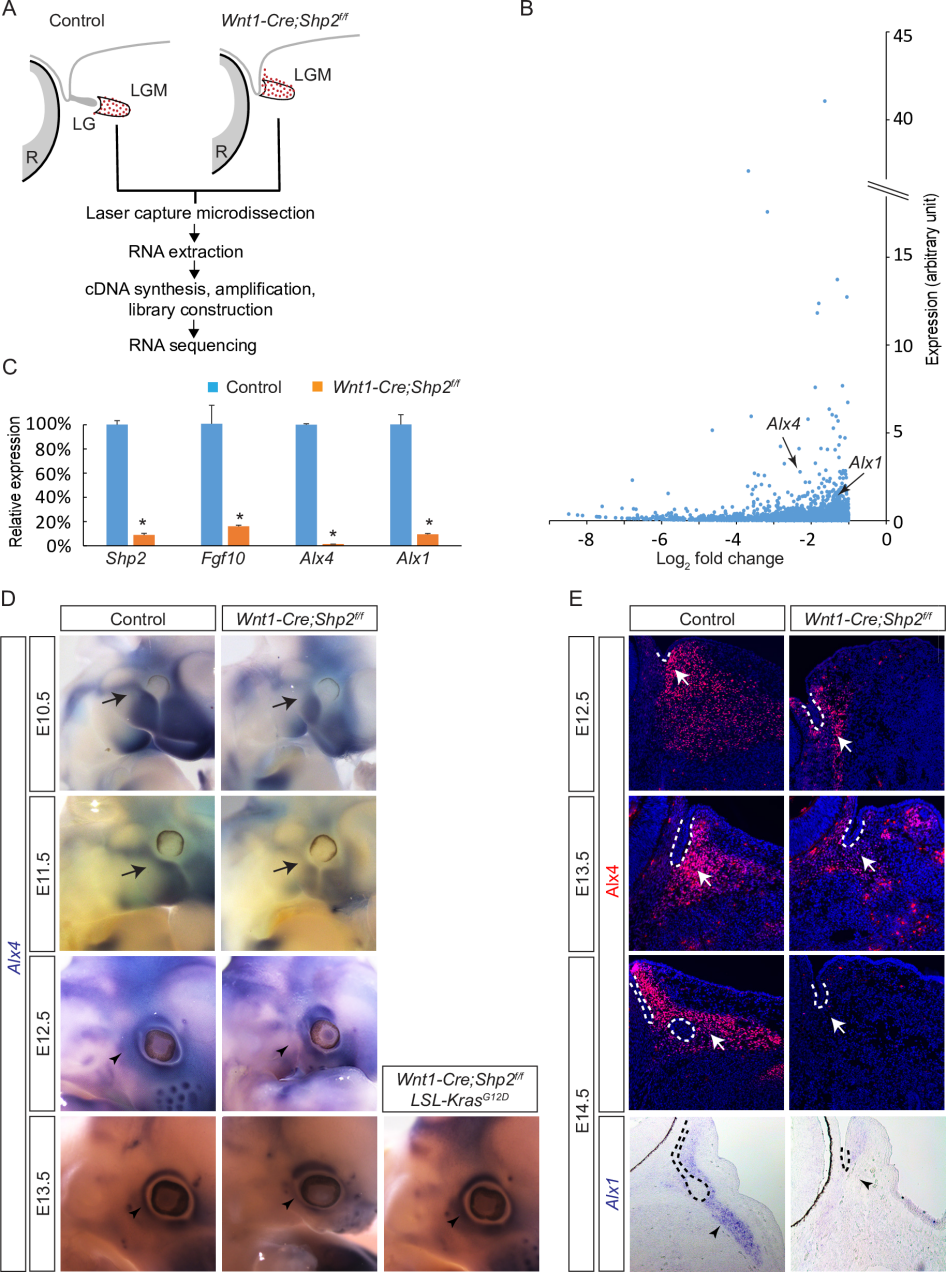
Identification of Alx genes downstream of Shp2 signaling in lacrimal gland development. (**A**) Schematic diagram of laser capture microscopy to isolate the periocular mesenchyme for RNA-seq analysis. (**B**) Dot plot of genes downregulated at least two folds in the *Shp2* mutants. The *Alx1* and *Alx4* genes are marked by arrows. (**C**) qRT-PCR confirmed the deletion of *Shp2* and down regulation of *Fgf10*, *Alx1* and *Alx4* in the laser captured periocular mesenchyme from *Shp2* mutants. Student’s *t* test: *P<0.001, *n* = 3. (**D**) *Shp2* deletion reduced *Alx4* expression in the cranial mesenchyme, especially at the periocular region next to the future lacrimal gland at E13.5, which was ameliorated in *Wnt1-Cre;Shp2*^*f/f*^*;LSL-Kras*^*G12D*^ embryos. Arrow: *Alx4* expression in the cranial mesenchyme at E10.5 and E11.5. Arrowhead: *Alx4* expression in the periocular mesenchyme at E12.5 and E13.5. (**E**) In *Shp2* mutants, Alx4 was progressively reduced in the periocular mesenchyme adjacent to the conjunctival epithelium from E12.5 to E13.5. By E14.5, both *Alx1* and Alx4 were lost. Arrow: Alx4 immunostaining in the periocular mesenchyme. Arrowhead: *Alx1* expression surrounding the lacrimal gland bud. Lacrimal gland primordia are outlined in dotted lines.

To determine the molecular basis of the lacrimal gland defect observed in *Shp2* mutants, we isolated the E14.5 periocular mesenchyme via laser capture micro-dissection and subsequently performed RNAseq analysis (Fig 5A). Among genes that were downregulated at least two folds in *Shp2* mutants, the third and eighth most highly expressed transcription factors were *Alx4* and *Alx1*, respectively (Fig 5B). These results were confirmed by a qPCR analysis of micro-dissected tissues, which also showed significant reductions in *Shp2* and *Fgf10* expressions as expected (Fig 5C).

We next focused on *Alx4* and *Alx1* as downstream targets of Shp2 signaling. At both E10.5 and E11.5, *Alx4* was widely expressed in the cranial mesenchyme surrounding the wild-type eye, but the expression was moderately reduced in *Shp2* mutants (Fig 5D, arrows). At E12.5, a more pronounced reduction of *Alx4* expression was evident at the temporal side of the mutant eye, where the lacrimal gland bud would have normally emerged. By E13.5, *Alx4* expression was absent in all areas of the periocular region except the dorsal side, but recovered in *Wnt1-Cre;Shp2*^*f/f*^*;LSL-Kras*^*G12D*^ embryos (Fig 5D, arrowheads). Immunostaining on sections further confirmed that *Shp2* deletion led to a progressive down regulation of Alx4 in the periocular mesenchyme, until it was entirely lost by E14.5 (Fig 5E, arrows). Similarly, *Alx1* in control wild-type embryos was expressed just anterior to the elongating lacrimal gland bud at E14.5, but this domain of *Alx1* expression eventually vanished in *Shp2* mutant embryos (Fig 5E, arrowheads). These results demonstrate that the periocular expressions of both *Alx1* and *Alx4* are regulated by Shp2 signaling.

### Alx4 binds a terrestrially conserved *Fgf10* genomic element to regulate its expression in the lacrimal gland mesenchyme

**Fig 6:**
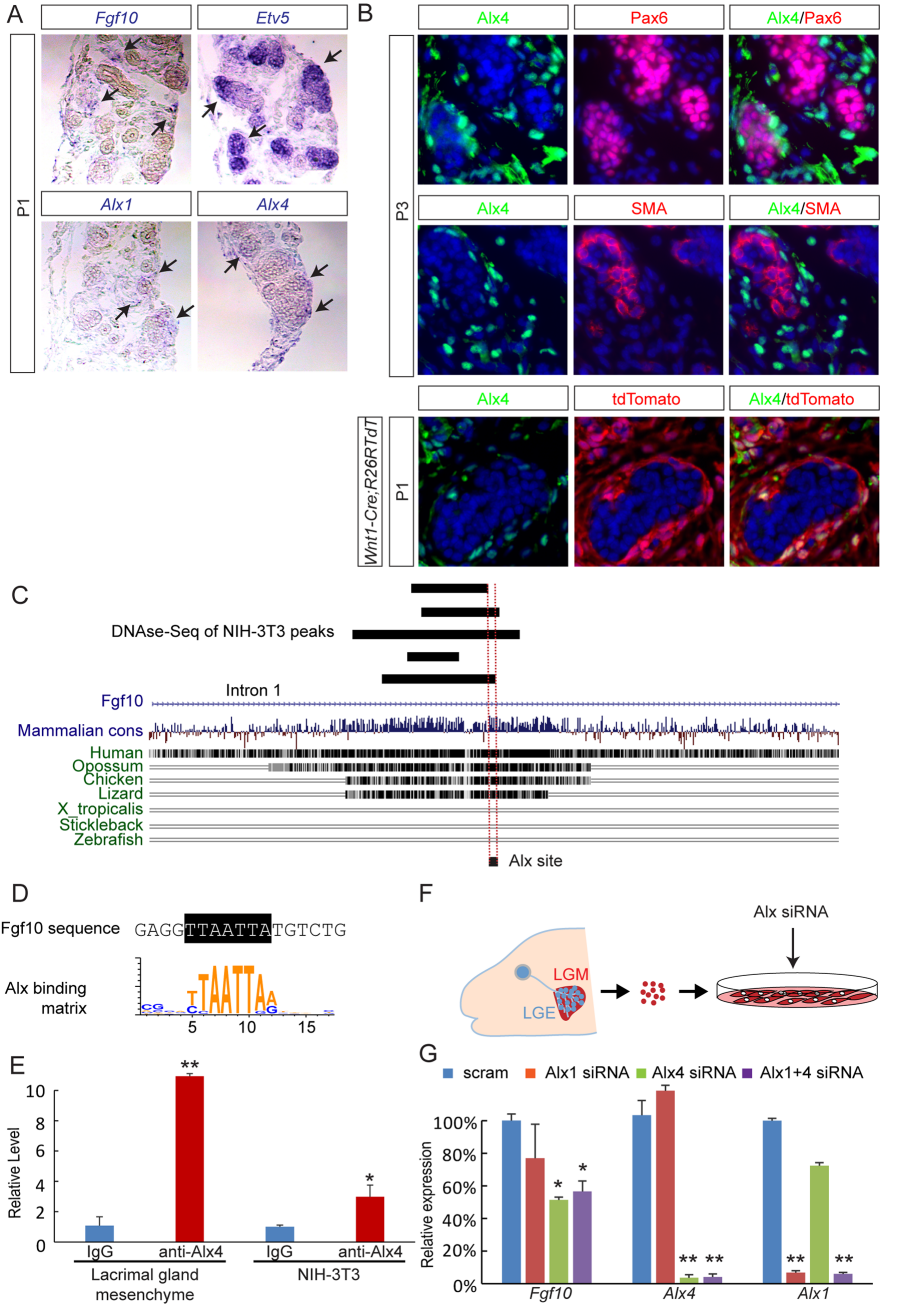
Alx4 binds a terrestrially conserved element in the *Fgf10* locus. (**A**) In new born pups, *Alx1*, *Alx4* and *Fgf10* were expressed in the lacrimal gland mesenchyme, whereas the FGF response gene *Etv5* was expressed in the epithelium. (**B**) Alx4 was excluded from Pax6-positive epithelial cells and SMA-positive myoepithelial cells, but it was expressed in the neural crest derived mesenchymal cells labeled by *Wnt1-Cre* induced tdTomato fluorescence. (**C**) Sequence alignment identified an Alx4 site within an intronic region of *Fgf10*, which was conserved from human to lizard, but not in species ranging from Xenopus to fish. It resided next to DNase hypersensitivity peaks in NIH3T3 cells. (**D**) The Alx4 site in the *Fgf10* locus matched the Alx consensus sequence. (**E**) Chromatin immunoprecipitation showed that Alx4 directly bound the Fgf10 intronic site in both lacrimal gland mesenchyme and NIH3T3 cells. Student’s *t* test: *P<0.01, *n* = 4; **P<0.001, *n* = 3. (**F**) Schematic diagram of mesenchymal cell culture isolated from newborn pups and treatment with *Alx* siRNA. LGM: lacrimal gland mesenchyme. LGE: lacrimal gland epithelium. (**G**) *Alx4* siRNA significantly down regulated *Fgf10* expression in lacrimal mesenchymal cells, whereas additional application of *Alx1* siRNA did not lead to further reduction. One Way ANOVA: *P<0.01, **P<0.001, *n* = 3.

The results above revealed a close resemblance of *Alx1* and *Alx4* expressions in the periocular mesenchyme to that of *Fgf10* during embryonic development. To evaluate this further, we examined their expression patterns in the neonatal lacrimal gland. At postnatal day 0 (P0), *Fgf10* was detectable in the mesenchymal cells, whereas the FGF-inducible gene *Etv5* was expressed in the adjacent ducts and acini, suggesting that FGF signaling remained active at this stage (Fig 6A, arrows). As expected, both *Alx1* and *Alx4* mRNA were also found in the lacrimal gland mesenchyme. Through immunostaining, we further demonstrated that the P3 lacrimal gland expressed the Alx4 protein, which was separated from both the epithelial marker Pax6 and the myoepithelial marker SMA (Fig 6B). Finally, to trace the origin of these Alx4-expressing cells in the lacrimal gland, we crossed *Wnt1-Cre* with an *R26TdT* (*Ai14*) reporter to indelibly label the neural crest-derived cells with tdTomato fluorescence. We then confirmed through immunostaining that Alx4 resided exclusively in the tdTomato-positive cells, demonstrating that Alx4 persisted in the neural crest lineage throughout lacrimal gland development.

Based on the similarities observed between *Alx1/4* and *Fgf10* expression patterns during lacrimal gland development, we hypothesized that Alx1 and Alx4 were direct regulators of *Fgf10* transcription. Because formation of the lacrimal gland was an adaptation of terrestrial animals to an airy environment, we searched the *Fgf10* locus for regions that were evolutionarily conserved from human to chicken but not in stickleback fish (Fig 6C). We next overlaid these regions with DNase hypersensitive sites in a 3T3 fibroblast cell line identified by the ENCODE project, because this cell line expressed both *Alx4* and *Fgf10* at high levels [33]. Finally, we screened these sequences using the Alx1/3/4 binding motif and identified a perfect match within intron 1 of *Fgf10* (Fig 6D). Interestingly, sequence alignment showed that this site was evolutionarily conserved among reptiles that have the lacrimal gland, such as the lizard, but not in Xenopus frog, which lacks one (Fig 6C) [34].

To ascertain whether this sequence was a bona fide Alx binding site, we performed chromatin immunoprecipitation in 3T3 cells followed by qPCR using specific primers. Compared to the IgG control, there was a ~3 fold enrichment of this putative Alx binding element in chromatins pulled down by the Alx4 antibody (Fig 6E). This was further validated in vivo by Alx4 chromatin immunoprecipitation using the lacrimal gland mesenchyme isolated from neonatal pups, which resulted in a ~11 fold enrichment. We next knocked down *Alx1* and *Alx4* using siRNAs in cultured lacrimal gland mesenchymal cells (Fig 6F). Interestingly, *Alx1* depletion led to a modest reduction in *Fgf10* mRNA levels, but the effect was not statistically significant (Fig 6G). In contrast, the *Alx4* knockdown decreased Fgf10 expression by ~50%, which was not further reduced by the combined treatment of both *Alx1* and *Alx4* siRNAs. This result suggested that *Alx4* plays a more dominant role than *Alx1* in regulating *Fgf10* within the lacrimal gland mesenchyme.

### Alx4 is required for lacrimal gland development in mouse and human

**Fig 7:**
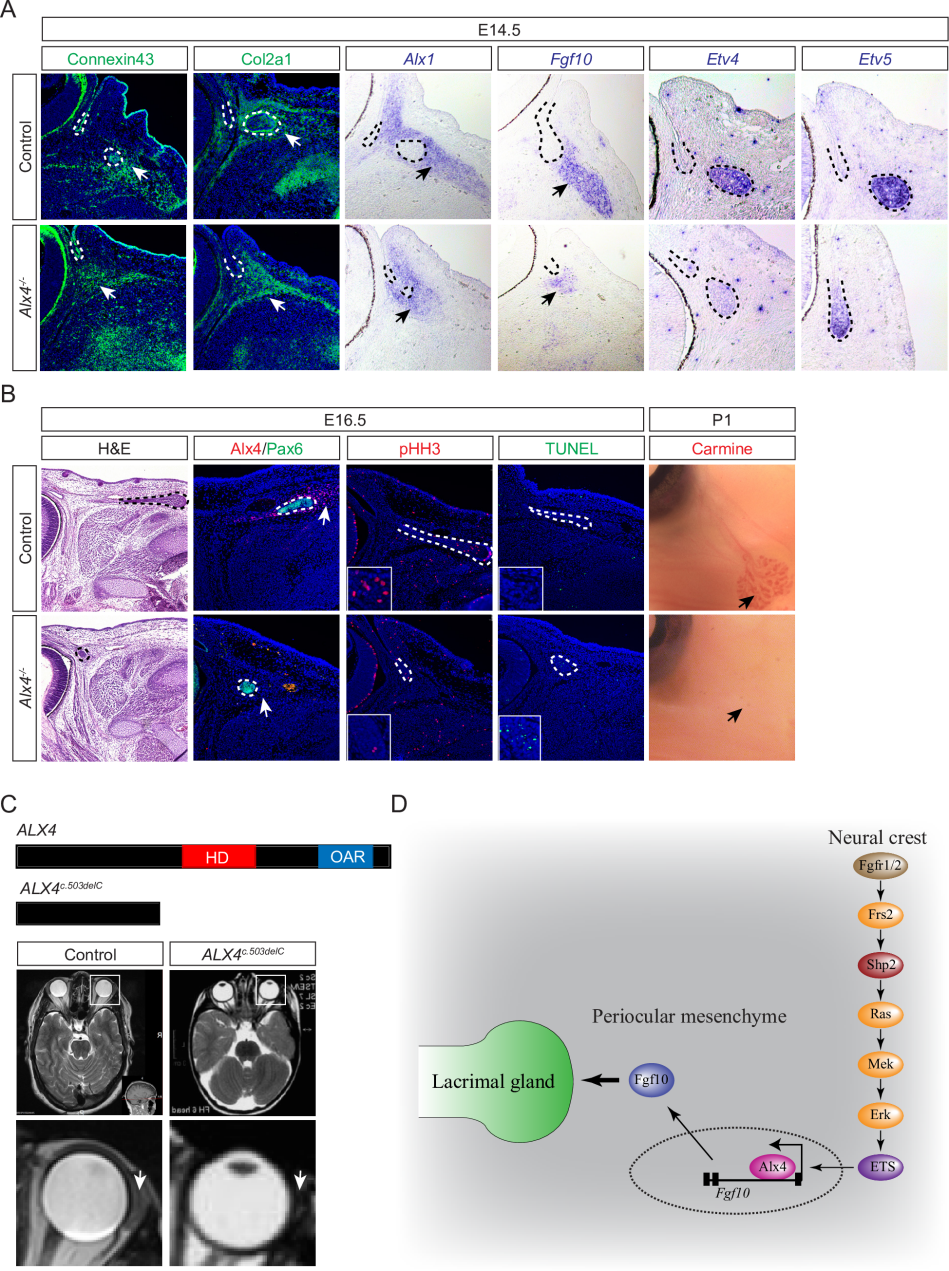
Alx4 inactivation led to lacrimal gland aplasia in human and mouse. (**A**) In E14.5 *Alx4* knockout embryos, Connexin43 and Col2a1 expression remained in the periocular mesenchyme, whereas the *Alx1* expression domain was reduced. *Fgf10*, *Etv4* and *Etv5* were significantly downregulated. Arrows: staining in the periocular mesenchyme. Lacrimal gland buds are outlined in dotted lines. (**B**) E16.5 *Alx4* null mutants merely displayed a rudimentary lacrimal gland shown by histology and Pax6 staining, while Alx4 immunostaining was lost altogether. There was a reduction of pHH3 and an increase in TUNEL staining within the residual bud (Inserts showed magnified region of lacrimal gland buds). At P1, carmine staining revealed an absence of the lacrimal gland in *Alx4* null pups. Lacrimal gland buds are outlined in dotted lines. (**C**) An MRI revealed the bilateral absence of the lacrimal gland in a patient carrying the c.503delC mutation that removed the functional domains of ALX4. Lower panel showed enlarged region of the eye and arrows point to the lacrimal gland. (**D**) Model of neural crest Shp2 signaling in lacrimal gland development. Shp2 mediates FGF signaling in the developing neural crest to activate Ras-MAPK signaling, which is required for Alx4 expression in the periocular mesenchyme. By binding to an intronic element of *Fgf10*, Alx4 activates Fgf10 expression to induce lacrimal gland budding.

To determine the functional role of Alx4 in lacrimal gland development, we analyzed *Alx4*^*lst-J*^ mice, which carried a frameshift mutation that removed both the homeodomain and downstream CAR domain. Homozygous *Alx4*^*lst-J*^ animals displayed craniofacial defects, dorsal alopecia and preaxial polydactyly at birth as previously reported in *Alx4* knockouts [35, 36]. At E14.5, *Alx4*^*lst-J*^ homozygous embryos maintained normal expression levels of Connexin43 and Col2a1 in the periocular mesenchyme, but the domain of *Alx1* expression was more restricted (Fig 7A, arrows). Importantly, there was a drastic reduction of *Fgf10* adjacent to the lacrimal gland bud, accompanied by a downregulation of FGF-target genes *Etv4* and *Etv5* in the lacrimal gland epithelium (Fig 7A, dotted lines). At E16.5, histology and immunostaining revealed a complete loss of Alx4 expression in the periocular mesenchyme and a much shorter Pax6-expressing lacrimal gland bud, characterized by reduced phospho-Histone H3 (pHH3) and increasing TUNEL signal (Fig 7B, dotted lines). By P1, no lacrimal gland was detectable by Carmine staining in *Alx4*^*lst-J*^ homozygous pups (Fig 7B, black arrows). These results demonstrated that inactivation of *Alx4* markedly disrupted Fgf10 expression and downstream FGF signaling, affected cell proliferation and survival, and ultimately caused a failure of lacrimal gland development.

In human, *ALX4* loss-of-function mutations underlie autosomal recessive frontonasal dysplasia 2 syndrome, characterized by skull defects, wide nasal bridge, notched nares, depressed nasal tip, hypertelorism and alopecia (OMIM 613451). We reanalyzed one patient carrying a homozygous c.503delC mutation in exon 2 of the *ALX4* gene, which resulted in the truncation of the homeobox (HD) and C-terminal OAR domains [37]. MRI imaging in that patient revealed a bilateral absence of lacrimal glands (Fig 7C, arrows). The patient lacked tearing and experienced irritable eyes and multiple episodes of eye infection since birth. This finding is consistent with the role of *ALX4* in human lacrimal gland formation.

## Discussion

In this study, we show that FGF signaling in the neural crest is required for *Fgf10* production within the periocular mesenchyme, thereby triggering a second round of FGF signaling in the conjunctival epithelium to form the lacrimal gland (Fig 7D). This is mediated by Frs2 and Shp2, which together activate the Ras-MAPK pathway to control the survival, migration and differentiation of the cranial neural crest cells. The downstream effector of Shp2 signaling in the periocular mesenchyme is the homeodomain transcription factor Alx4, which binds a terrestrially conserved element to regulate *Fgf10* expression in the periocular mesenchyme, reflecting the evolutionary history of the lacrimal gland. Our results highlight the sequential use of FGF signaling in neural crest development and reveal the etiology of lacrimal insufficiency in an *ALX4* patient.

RASopathies represent a spectrum of congenital abnormalities caused by aberrant Ras-MAPK signaling, but the particular RTK signaling pathway mediated by Ras in the normal development of a specific tissue is not always clear [38, 39]. Using mouse genetics, we showed that defective FGF signaling, and not PDGF signaling, in the neural crest reproduced the Shp2 conditional knockout phenotype seen in the lacrimal gland, thereby positioning FGF receptors as the primary regulators of Shp2 function in the neural crest cells that partake in directing the development of the lacrimal gland. Contrary to a previous study in zebrafish, we did not observe that Shp2 acts upstream of p53 to suppress neural crest cell apoptosis [26]. This discrepancy could be due to differences either intrinsic to the species used or to the experimental approaches utilized as we took advantage of conditional knockouts in mice whereas the zebrafish study used a morpholinos knockdown. Instead, our genetic evidence demonstrates a fundamental role for the Shp2-Ras-Mek-Erk signaling cascade in neural crest survival and development. MAPK is known to phosphorylate and induce the ETS domain transcription factors, which act as downstream effectors in gene regulation. In particular, the expressions of *Pea3* family genes *Etv1/4/5* correlate closely with FGF signaling activities during embryonic development [10]. While deletion of all three *Pea3* family genes in the neural crest failed to produce any craniofacial or lacrimal gland defects, the overexpression of a dominant-negative *Etv4* lead to stunted lacrimal gland growth. This suggests that other members of the ETS domain transcription factors, which recognize similar binding sites as Etv1/4/5, can play redundant roles in transmitting FGF-MAPK signaling during neural crest development.

Our study demonstrates that *Alx* genes are the ultimate downstream effectors of Shp2 signaling in the periocular mesenchyme. Alx4 shares both sequence and structural homologies of paired-type homeodomain and C-terminal aristaless domain with two other transcription factors, Alx1 and Alx3. These proteins are present within the craniofacial mesenchyme and limb bud, displaying overlapping expression patterns [40]. Members of this family of transcription factors also exhibit functional redundancies as shown by genetic interactions in specific tissues. *Alx3* knockout mice were morphologically normal, but *Alx3/4* double mutants displayed more severe defects in the neural crest-derived craniofacial structures than the *Alx4* knockout alone [40]. *Alx1* null mice showed craniofacial defects distinct from *Alx4* mutants and combined deletion of both genes led to developmental abnormalities not found in either of the single mutants, indicating that *Alx1* and *Alx4* have both unique and redundant roles [36]. The lacrimal gland mesenchyme expresses *Alx1* and *Alx4*, but not *Alx3*. Although we did not observe a synergistic effect of *Alx1* and *Alx4* in our in vitro experiments, it remains possible that *Alx4*/*Alx1* double knockout mice will present comparably severe lacrimal gland defects as the neural crest *Shp2* mutant did.

The precise level of *FGF10/Fgf10* expression in the periocular mesenchyme is critical for lacrimal gland induction. This is clearly shown by aplasia of the lacrimal and salivary glands (ALSG) and Lacrimo*-*auriculo*-*dento*-*digital (LADD) syndromes, in which even heterozygous mutations in human *FGF10* can lead to congenital lacrimal gland defects [41, 42]. Our study has demonstrated that neural crest FGF signaling is required for *Fgf10* expression in the periocular mesenchyme, but the ligand of the neural crest FGF signaling that leads to lacrimal gland development remains an open question. It is unlikely to be the autocrine signaling of Fgf10, because deletion of Fgfr2, the cognate receptor for Fgf10, in the neural crest only produced minor defects in lacrimal gland development (Fig 1). In limb development, the mesenchyme-derived Fgf10 signals the epithelium to induce Fgf8 and later Fgf4, Fgf9 and Fgf17, which in turn act on Fgfr1 and Fgfr2 in the mesenchyme to maintain Fgf10 expression [43–45]. During lung development, Fgfr1 and Fgfr2 in the mesenchyme respond to Fgf9 expressed by the lung epithelium and mesothelium. This maintains the mesenchymal expression of Fgf10 that signals back to the epithelium [46, 47]. Submandibular salivary gland development is yet another example where the epithelium-mesenchyme interaction plays an important role. In this case, Fgf10 in the mesenchyme originated from the cranial neural crest is modulated by ectodermal-derived Fgf8 [48]. However, neither a systemic knockout of *Fgf9* nor deletion of *Fgf8* using Cre transgenes specific to the midbrain-hindbrain junctiondisrupt lacrimal gland development (S3 Fig and Fig 4A). Considering the complexity of the FGF family, further work is needed to identify the relevant FGF ligands for the neural crest FGF signaling pathway during lacrimal gland development.

The main and accessory lacrimal glands secrete the aqueous component of the tear film, and thereby play an important role in maintaining the health and transparency of the ocular surface. Because the tear is only necessary for land animals whose eyes are constantly exposed to the air, the lacrimal gland emerged relatively late in the evolution of the vertebrate tetrapod. Even among animals living both on land and in water, the lacrimal gland is only present in reptiles such as the alligator, but not in amphibians such as the frog (S4 Fig). In this study, we show that the *Alx4* binding site in the *Fgf10* locus lies within a region that’s conserved from humans to alligators, but not in frogs or fish. This suggests that, although both *Alx4* and *Fgf10* arose in more primitive organisms, these two genes were most likely not functionally linked until the emergence of the lacrimal gland in reptiles. Considering that *Fgf10* lies at the top of the genetic cascade for inducing branching morphogenesis in many glandular organs, this represents an example of evolution that coopts an existing genetic circuitry to develop new organs that enable the adaptation to new environments. By showing that the Alx4-Fgf10 axis is conserved from mouse to human, our study contributes to the understanding of the role of Alx4 in human neural crest cell and lacrimal gland development and points in the direction of generating the lacrimal gland from pluripotent stem cells.

## Materials and methods

### Ethics statement

The animal experiments were approved by Columbia University Institutional Animal Care and Use Committee (IACUC).

### Mice

Mice carrying *Erk1*^*-/-*^, *Erk2*^*flox*^, *Frs2α*^*flox*^, *Frs2α*^*2F*^, *Mek1*^*flox*^, *Mek2*^*KO*^, *Shp2*^*flox*^ alleles were bred and genotyped as described [20, 49–52]. We obtained *Etv1*^*flox*^ mice from Dr. Silvia Arber (University of Basel, Basel, Switzerland), *Etv4*^*-/-*^ and *Etv5*^*flox*^ mice from Dr. Xin Sun (University of California at San Diego, San Diego, CA), *En1-Cre* and *R26-EtvEnR* from Dr. James Li (University of Connecticut Health Center, Farmington, CT), *Fgf8*^*flox*^ from Dr. Suzanne Monsour (University of Utah, Salt Lake city, UT), *Fgf15*^*-/-*^ from Dr. Steven Kliewer (UT Southwestern Medical Center, Dallas, TX), *Fgfr1*^*ΔFrs*^ from Dr. Raj Ladher (RIKEN Kobe Institute-Center for Developmental Biology, Kobe, Japan), *Fgfr2*^*LR*^ from Dr. Jacob V.P. Eswarakumara (Yale University School of Medicine, New Haven, CT) and *Fgf9*^*-/-*^ and *Fgfr2*^*flox*^ from Dr. David Ornitz (Washington University Medical School, St Louis, MO) [16, 19, 28, 53–58]. *LSL-Kras*^*G12D*^ mice was obtained from the Mouse Models of Human Cancers Consortium (MMHCC) Repository at National Cancer Institute [59]. *Alx4*^*lst-J*^ (Stock No: 000221), *Fgfr1*^*flox*^ (Stock No: 007671), *p53*^*flox*^ (Stock No: 008462), *Pdgfr*α^*flox*^ (Stock No: 006492), *R26R* (Stock No: 003474), *R26RTdT* (*Ai14*, Stock No: 007914), *Sox10-Cre* (Stock No: 025807) and *Wnt1-Cre* (Stock No: 009107) mice were obtained from Jackson Laboratory [16, 40, 60–64]. Animals were maintained on mixed genetic background. *Wnt1-Cre* or *Shp2*^*flox*^ only mice did not display any lacrimal gland phenotypes and were used as controls.

### Histology and immunohistochemistry

Histology, carmine staining, TUNEL assays and immunohistochemistry are performed as previously described [11, 65]. The following primary antibodies were used: Alx4 (sc-33643, Santa Cruz Biotechnology), E-cadherin (U3254, Sigma, St Louis, Missouri), Cleaved-caspase 3 (#9664, Cell signaling Technology), Col2a1 (ab34712, Abcam), Connexin43 (#3512, Cell signaling Technology), pHH-3 (#06–570, Millipore), Pax6 (PRB-278P, Covance, Berkeley, CA, USA), RFP (#600-401-379, Rockland), α-SMA (#C6198, Sigma-Aldrich).

### X-gal staining

E13.5 embryos were incubated in 4% PFA for 1 hr at 4°C and washed twice in PBS containing 0.02% NP-40, 0.01% sodium deoxycholate and 2 μg/ml MgCl_2_ for 30 min each, followed by overnight incubation in X-gal staining solution (1 mg/ml X-gal, 10 mM Potassium Ferricyanide, 10nM Potassium Ferrocyanide, 2 μg/ml MgCl_2_ in PBS) at 4°C. The samples were then cryopreserved in OCT (Sakura Finetek), sectioned at 10 μm thickness and counter-staining with nuclear red.

### RNA in situ hybridization

RNA in situ hybridization was performed as previously described [66]. The following probes were used: *Alx1* (from Dr. Terence Capellini, Harvard University, Boston, MA), *Alx4* (from Dr. Yang Chai, University of Southern California, Los Angeles, CA), *Etv4*, *Etv5* (from Dr. Bridget Hogan, Duke University Medical Center, Durham, NC, USA), *Foxc1* (from Dr Anthony Firulli, Indiana University School of Medicine, Indianapolis, IN, USA), *Fgf10* (for whole mount) (from Dr. Suzanne Monsour, University of Utah, Salt Lake city, UT)), *Fgf10* (for sections)was generated from a full length cDNA clone (IMAGE: 6313081 Open Biosystems, Huntsville, AL, USA), *Pitx2* (from Dr. Valerie Dupé, CNRS, Strasbourg, France).

### Laser capture micro-dissection and gene expression profiling

Freshly harvested embryos were frozen in the OCT, sectioned at 10μm thickness and transferred to PEN slides (Zeiss). Slides were dipped in 95% ethanol for 2 min to fix the samples and stained with crystal violet stain (3% in ethanol) on ice. This was followed by dipping in 70% ethanol for 30–40 sec to remove the OCT and dehydration in 100% ethanol for 2 min. The periocular mesenchymal tissue was micro-dissected using Laser capture microscope (Zeiss AxioObserver.Z1 inverted microscope). 500 pg of RNA was isolated from each sample, converted to cDNA and amplified using Nugen Ovation kit (Nugen) to obtain 2–3 μg cDNA, which was then converted to cDNA library for RNA-sequencing analysis at core facility in Columbia University. The RNAseq data is available at the GEO repository under accession number GSE103402.

### Lacrimal gland mesenchyme culture

Lacrimal glands mesenchymal culture was performed as described previously [67]. Briefly, glands were isolated from P0-P2 pups and trypsinized (Gibco 1:250) at 4°C for 1 hr. After neutralizing trypsin, the mesenchyme was manually separated from the epithelium using fine needle and grown in the complete medium (DMEM+10% FBS with antibiotics) for 3 days before passage. The primary mesenchymal cells were transfected with siRNA using Lipofectamine RNAimax as previously described and harvested after 24–48 hrs [68]. For *Alx1* and *Alx4*, the results were confirmed using two different predesigned Silencer® Select siRNAs from Ambion (Life technologies).

### Quantitative-PCR (qPCR)

Quantitative-PCR was performed as described [69]. Primer sequences used were, *Alx4*: 5’-ACACATGGGCAGCCTGTTTG3’, 5-TGCTTGAGGTCTTGCGGTCT-3’, *Alx1*: 5’ GGAGGAAGTGAGCAGAGGTG-3’, 5’- TTCAAATGCGTGTCCGTTGGT-3’, *Fgf10*: 5’ CAATGGCAGGCAAATGTATG-3’, 5’- GGAGGAAGTGAGCAGAGGTG-3’, *Gapdh*: 5’-AGGTCGGTGTGAACGGATTTG-3’, 5’-TGTAGACCATGTAGTTGAGGTCA-3’, *Shp2* (exon 4): 5’- CTGACGGAGAAGGGCAAGCA-3’, 5’- CGCACGGAGAGAACGAAGTCT-3’.

### Chromatin immunoprecipitation

The Chromatin Immunoprecipitation (ChIP) assays were performed in 3T3 fibroblasts cells and primary lacrimal gland mesenchymal cells as described [70]. Briefly, the cells grown in DMEM/10% FBS with antibiotics were crosslinked with 1% Formaldehyde for 8–10 min with gentle shaking. This was followed by quenching with 125 mM glycine or 5 min, 3X washing with cold PBS and addition of 1 ml of cold CHIP lysis buffer. After incubation for 10 min at 4°C, the lysed cells were centrifuged at 3000 rpm for 3 min and the pellet were stored at -80°C until later use. The pellet was then resuspended in 1.2 ml of RIPA buffer, sonicated on ice for 8 min using probe sonicator (1 sec “on”, 2 sec “off”, power 3.5) and centrifuged at 13000 rpm for 15 min at 4°C. The supernatant was precleared by adding 45 μl Protein G agarose beads (50% slurry, Millipore) and incubated for 2 hrs at 4°C on rotor. After centrifugation at 5000 rpm for 1 min, the supernatant was transferred to a fresh tube and the protein concentration was measured by Bradford assay. For pull down, 1 μg of antibodies were added per 1mg of protein for overnight incubation at 4°C, followed by addition of 20 μl agarose beads for another 1–2 hours incubation. After brief centrifugation, the beads were washed 1X with RIPA buffer at room temperature, 3X with cold RIPA buffer, 2X with cold Wash buffer A and Wash buffer B, 1X with TE/150mM NaCl. Next, the samples were decrosslinked in Elution buffer containing RNAase (40μg/ml) and Proteinase K (20μg/ml) for 1 hr at room temperature and 50°C overnight. After brief centrifugation, the supernatant was treated with equal vol. of Phenol/Chloroform and the DNA was precipitated with 2.5 vol. of 100% ethanol and Glycoblue for 1 hr at -80°C and dissolved in 20 μl sterile water for qPCR analysis. The antibodies used were IgG as isotype control (sc-2028, Santa Cruz Biotechnology) and anti-Alx4 (sc-22066, Santa Cruz Biotechnology). *Buffer recipes*: CHIP lysis buffer- 10mM Tris-Cl, pH8, 85mM KCl, 0.5% NP-40, 5nM EDTA, 0.25% Triton; RIPA- 1% Triton, 150mM NaCl, 0.1% SDS, 0.1% Na-Deoxycholate, 10mM Tris-Cl, pH8, 5mM EDTA; Wash buffer A- 50mM HEPES, pH7.9, 500mM NaCl, 1mM EDTA, 1% Triton, 0.1% Na-deoxycholate, 0.1% SDS, Wash buffer B- 20mM Tris-Cl, pH8, 1mM EDTA, 250 mM LiCl, 0.5% NP-40, 0.5% Na-deoxycholate; Elution Buffer- 1% SDS, 30 mM Tris-Cl (pH8), 15mM EDTA, 200mM NaCl. Protease inhibitor cocktail is added prior to use in all the buffers until ready to elute. The primers used for CHIP in intron 1 of Fgf10- F- 5’-GGTTGGAGCTTGTTGTGTGT-3’, R- 5’-GCTCTGCTAATAAAGGTCTCCC-3’.

### Bioinformatics analysis

We retrieved 200 KB upstream and 100 KB downstream of *Fgf10* transcription start site from Mouse Genome assembly GRCm38/mm10 and analyzed this sequence for evolutionary conservation using UCSC genome browser. These sequences were also overlaid with the DNase-hypersensitivity data from 3T3 cell line retrieved from ENCODE database and scanned for Alx4 consensus binding sites based on TRANSFAC (release 2013.1) database using MATCH algorithm, with minFP as parameter to identity sites with minimum false positives.

## Supporting information

S1 Fig Frs2-Shp2 interaction is required for lacrimal gland development. In the *Wnt1-Cre;Frs2*^*f/2F*^ mutant that disabled Shp2 binding to Frs2, lacrimal gland development was aborted at E14.5 (*n* = 6).
(PDF)

S2 Fig Shp2 deletion in the migratory neural crests disrupted lacrimal gland development. (**A**-**B**) *Sox10-Cre* mediated ablation of *Shp2* in the migrating neural crest also abolished lacrimal gland budding at E14.5 (arrows). (**C-D**) *Fgf10* expression was lost in the periocular mesenchyme (arrowheads). Lacrimal gland primordia are outlined with dotted lines.
(PDF)

S3 Fig *Fgf9* knockout did not affect lacrimal gland development. *Fgf9*^*-/-*^ embryo has the lacrimal gland (outlined in yellow dotted line).
(PDF)

S4 Fig Evolutionary conservation of the Alx4 site in the avian and reptile genome. The Alx4 binding region within the Fgf10 locus is conserved in species ranging from the finch to the lizard.
(PDF)
