# Supplementary figures and images for: Alx4 relays sequential FGF signaling to induce lacrimal gland morphogenesis
Source: PLoS Genet. 2017 Oct 13;13(10):e1007047. doi: 10.1371/journal.pgen.1007047 (PMC5656309; doi:10.1371/journal.pgen.1007047)

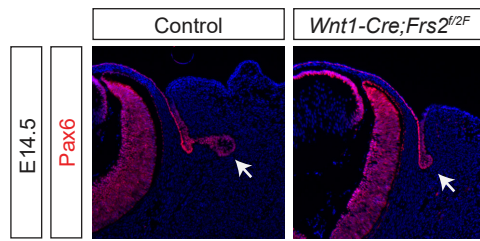

Supplement: S1 Fig — In the Wnt1-Cre;Frs2f/2F mutant that disabled Shp2 binding to Frs2, lacrimal gland development was aborted at E14.5 (n = 6). (PDF) [file pgen.1007047.s001.pdf]

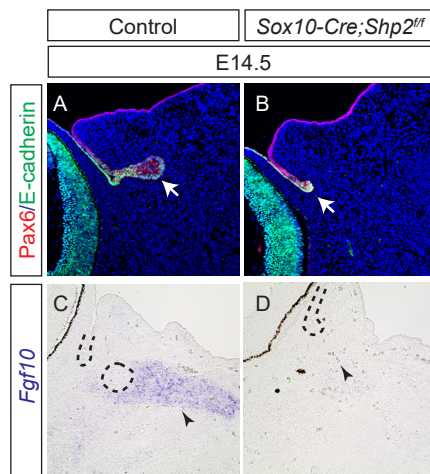

Supplement: S2 Fig — (A-B) Sox10-Cre mediated ablation of Shp2 in the migrating neural crest also abolished lacrimal gland budding at E14.5 (arrows). (C-D) Fgf10 expression was lost in the periocular mesenchyme (arrowheads). Lacrimal gland primordia are outlined with dotted lines. (PDF) [file pgen.1007047.s002.pdf]

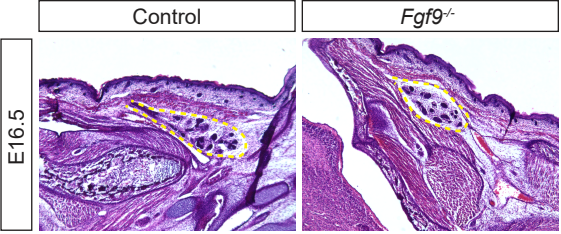

Supplement: S3 Fig — Fgf9-/- embryo has the lacrimal gland (outlined in yellow dotted line). (PDF) [file pgen.1007047.s003.pdf]

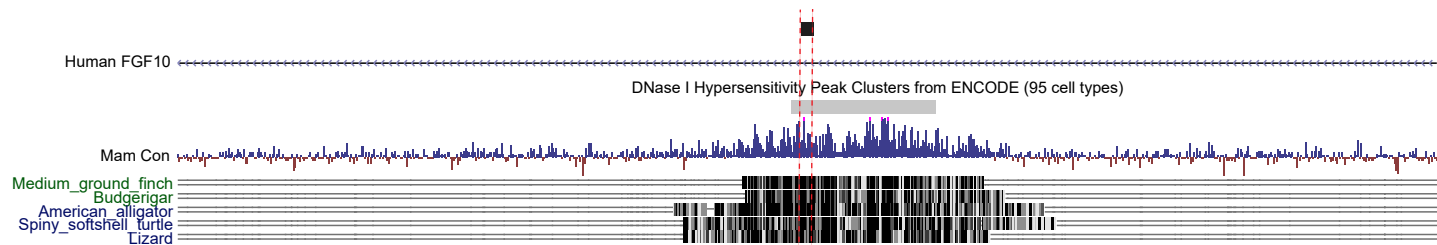

Supplement: S4 Fig — The Alx4 binding region within the Fgf10 locus is conserved in species ranging from the finch to the lizard. (PDF) [file pgen.1007047.s004.pdf]
